# Mechanisms of *Lycium barbarum* Treatment in Sepsis‐Induced Myocardial Injury: A Network Pharmacology and Experimental Validation Study

**DOI:** 10.1002/fsn3.72197

**Published:** 2026-08-12

**Authors:** Ruxin Liu, Chunxia Ma, Yujiao Tang, Xue Bai, Liting Yang, Yi Hu, Zesu Niu, Weiliang Xue, Ling Zhang, Mengfei Chen

**Affiliations:** ^1^ People's Hospital of Ningxia Hui Autonomous Region (The Third Clinical Medical College of Ningxia Medical University) Yinchuan China

**Keywords:** drug target, *Lycium barbarum* (LB), molecular docking, network pharmacology, sepsis‐induced myocardial injury (SIMI)

## Abstract

*Lycium barbarum* (LB) has long been recognized for its anti‐inflammatory and anti‐aging properties, with recent evidence suggesting protective effects against myocardial injury. However, its role in sepsis‐induced myocardial injury (SIMI) remains unclear. In this study, we explored the molecular mechanisms and active components of LB against SIMI using network pharmacology combined with experimental validation. Chemical profiling identified the main constituents of LB aqueous extract, and 45 active compounds along with 320 potential targets were predicted. Among these, 49 targets overlapped between LB and SIMI, primarily involved in apoptosis and inflammation. Molecular docking further validated the strong binding affinity of key compounds to these targets. Both in vitro (LPS‐stimulated HL‐1 cardiomyocytes) and in vivo (cecal ligation and puncture‐induced septic mice) experiments demonstrated that LB suppressed the expression and secretion of core inflammatory and apoptotic proteins, inhibited the MAPK signaling pathway in mouse myocardial tissue, and ultimately reduced myocardial injury. These findings suggest that LB holds promise as a potential therapeutic agent for SIMI.

## Graphical abstract

The chemical characterization of the LB extract confirmed the presence of polysaccharides and specific small‐molecule constituents (e.g., rutin, isoquercitrin). Network pharmacology analysis predicted that the bioactive components of LB were able to interact with core targets such as TP53, AKT1, and JUN, and molecular docking simulations suggested quercetin, the aglycone of rutin and isoquercitrin, as a key effector contributing to its cardioprotective role in SIMI. In vivo and in vitro experiments demonstrated that the LB aqueous extract treatment inhibits the MAPK signaling pathway, leading to attenuated cardiomyocyte apoptosis and reduced levels of the inflammatory cytokines IL‐6, IL‐1β, and TNF‐α.

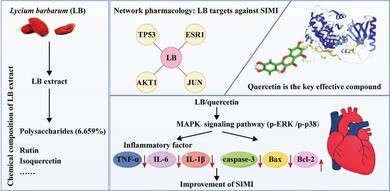

**Abbreviations**
BC: betweenness centrality
CC: closeness centrality
CCK‐8: cell counting kit 8
CLP: cecal ligation and puncture
DL: drug‐likeness
EC: eigenvector centrality
GO: gene ontology
KEGG: Kyoto encyclopedia of genes and genomes
LAC: local average connectivity
LB:
*Lycium barbarum*

LPS: lipopolysaccharide
OB: oral bioavailability
PPI: protein–protein interaction
RT‐qPCR: reverse transcription quantitative real‐time PCR
SD: standard deviation
SIMI: sepsis‐induced myocardial injury

## 1 Introduction

Sepsis is described as a deadly organic malfunction induced by dysregulation of the host response to infection (Hua et al. 2024). According to epidemiologic studies, approximately 19 million people suffer from severe sepsis and 5.3 million die each year globally, with the incidence increasing by 1.5%–8% per year (Gupta et al. 2023; Chen et al. 2025). Myocardial damage or cardiac insufficiency has been documented in 25%–50% of sepsis patients and is considered an essential pathognomonic marker of poor prognosis in septic patients (Li et al. 2024; Wang et al. 2023). If cardiac issues emerge, the patient's health rapidly deteriorates, and the mortality rate can be as high as approximately 70% (Du and Zhou 2023).

The clinical management of sepsis‐induced myocardial injury (SIMI) faces a dual dilemma: treatment failure caused by antibiotic resistance (such as polymyxin B) and an increase in secondary myocardial oxygen consumption induced by vasoactive drugs (Liu et al. 2017; Weiss and Fitzgerald 2024). New therapeutic approaches have been developed in the field of natural medicines as a result of this unmet clinical need (Zhang, Singla, et al. 2024). Based on the theory of “nourishing yin and strengthening the constitution, and harmonizing yin and yang” in traditional Chinese medicine, *Lycium barbarum* (LB) has become the focus of research due to its multi‐target antioxidant properties (Zhao et al. 2025). Experimental studies have shown that LB can alleviate doxorubicin‐induced acute cardiotoxicity by inhibiting the oxidative stress pathway and has demonstrated significant cardioprotective effects in the ischemia–reperfusion injury model (Pop et al. 2020; Liu et al. 2021). However, it is unknown whether LB is effective in the pathological scenario of SIMI, and its key molecular mechanisms and functions still need to be elucidated.

Network pharmacology is gaining popularity in the world of traditional Chinese medicine as a new research technique for investigating various pharmacological targets (Huang et al. 2023). In this study, we first constructed a bioactive compound‐target network of LB to predict its candidate targets against SIMI. The cytoprotective effect of LB was then evaluated in lipopolysaccharide (LPS)‐injured HL‐1 cardiomyocytes, followed by in vivo validation of its cardioprotective efficacy in a cecal ligation and puncture (CLP)‐induced septic mouse model. Finally, molecular docking simulations were performed to verify the interactions between key bioactive components and core targets. This integrated multi‐level approach provides comprehensive insight into the pharmacological basis of LB in ameliorating SIMI.

## 2 Materials and Methods

### 2.1 Data Collection and Processing

We collected the components in LB using the TCMSP (https://tcmspw.com/tcmsp.php), a methodical pharmacological database of Chinese medicines that contains 499 herbs in the 2010 version of the Chinese Pharmacopeia as well as the compound components of each herb (more than 29,000 in total) (Ru et al. 2014). We chose active compounds with oral bioavailability (OB) of 30% and drug‐likeness (DL) of 0.18. Forty five active chemical components of LB matched these inclusion requirements after a search and selection process (Zhang et al. 2025). Table S1 summarizes detailed information for each class of chemicals. The TCMSP database was used to identify proteins that are associated with the active components in LB.

### 2.2 Targets for Predicting SIMI

CLP‐induced sepsis myocardial injury rat microarray about SIMI‐related targets data were downloaded through the GEO database (GSE153086) (https://www.ncbi.nlm.nih.gov/geo/). And the WGCNA (Langfelder and Horvath 2008) to identify significant gene modules and determine relevance to sepsis myocardial injury rats. Two distinct databases, including the geneCards (https://www.genecards.org/) (Stelzer et al. 2016) and the National Center for Biotechnology Information (Brown et al. 2015). We used the phrase “myocardial injury in sepsis” and “myocardial damage in sepsis” to identify the common targets of GeneCards as sepsis myocardial injury‐related targets. To avoid leaving out established targets, 84 experimentally confirmed targets were acquired from Pubmed and added to the list to arrive at the final diabetic myocardial injury‐related targets.

### 2.3 Network Construction and Function Analysis

Cytoscape (http://www.cytoscape.org) was utilized to map the network correspondences of active drug and target of LB‐SIMI. String tool (https://string‐db.org/) was used to map protein–protein interaction (PPI) networks online, which were then imported into Cytoscape for visualization and presentation. To get the optimal balance between sensitivity and specificity, the confidence level was set at 0.7. MCODE clustered the PPI network into three primary modules, which were then examined by EnrichR (https://maayanlab.cloud/Enrichr/) (Bader and Hogue 2003) to determine the main executive function of each module.

Furthermore, the CytoNCA (Tang et al. 2015) pluginwas used to calculate protein–protein connectivity, including Betweenness (BC), Closeness (CC), Eigenvector (EC), and Local Average Connectivity‐based method (LAC), according to the criterion that all connectivity metrics exceeded the median value, as shown in Table 1. The “clusterProfiler” (Wu et al. 2021) R package for Gene Ontology (GO) and Kyoto Encyclopedia of Genes and Genomes (KEGG) pathway was utilized to evaluate the key potential targets.

**TABLE 1:** The main targets of LB treatment.

Acronym	Name	Betweenness	Closeness	Degree	Eigenvector	LAC	Network
TP53	Tumor protein P53	1330.7445	0.2588	54	0.3892	9.6296	35.2372
JUN	Jun proto‐oncogene	873.0412	0.2519	36	0.3144	11.3333	22.8186
AKT1	AKT serine/threonine kinase 1	584.3773	0.2481	32	0.2753	8.7500	15.2063
Bcl‐2	B‐cell lymphoma 2	334.2857	0.2418	28	0.2461	8.5714	15.0786
ESR1	Estrogen receptor 1	279.5029	0.2391	28	0.2644	10.0000	16.0114
MAPK1	Mitogen‐activated protein kinase 1	195.6832	0.2409	26	0.2670	10.769	15.1962
MYC	MYC proto‐oncogene	100.0779	0.2391	24	0.2613	11.0000	14.2416
CASP3	Caspase 3	564.5291	0.2349	22	0.1729	6.5455	10.9578
IL1B	Interleukin 1 Beta	655.4501	0.2245	20	0.1054	6.8000	10.5168
FOS	Fos proto‐oncogene	82.2221	0.2308	20	0.2083	8.8000	11.5370
RELA	RELA proto‐oncogene	156.7781	0.2340	18	0.1781	8.4444	9.7055
EGFR	Epidermal growth factor receptor	99.4550	0.2237	18	0.14534	4.8889	7.5548
HIF1A	Hypoxia inducible factor 1 subunit alpha	17.3215	0.2340	18	0.2330	11.1111	11.7647
CCL2	C‐C motif chemokine ligand 2	105.8043	0.2150	16	0.09042	8.5000	11.8626
CDK1	Cyclin dependent kinase 1	43.5537	0.2164	16	0.1070	5.5000	9.2260
CXCL8	C‐X‐C motif chemokine ligand 8	27.36790	0.2157	16	0.0949	8.5000	10.9229
Bcl‐2L1	Bcl‐2 like protein 1	59.8568	0.2284	14	0.1292	6.8571	9.34799
NFE2L2	Nuclear factor, erythroid 2 like 2	170.9000	0.2193	12	0.1048	4.6667	5.9636
GSK3B	Glycogen Synthase kinase 3 beta	130.0564	0.2292	12	0.1351	6.0000	6.5455
CASP8	Caspase 8	170.4318	0.2292	10	0.1036	4.8000	5.3333

### 2.4 Molecular Docking

Specific methods were used as described previously (Hausenloy et al. 2011). Briefly, the PubChem database (https://pubchem.ncbi.nlm.nih.gov/) was utilized to retrieve the ligands' two‐dimensional structures, which were then converted to three‐dimensional structures using Chem3D. Structures of receptor protein were obtained from the Protein Data Bank (PDB). Finally, molecular docking calculations were performed using Autodock Vina. The best affinity conformation was selected as the final docked conformation.

### 2.5 Preparation of Aqueous Extract of LB

500 g LB was purchased from Ningxia, China (lot 070101). LB was extracted with water in a 1:1 ratio (1 h × 3). The combined aqueous extracts were filtered, concentrated, and vacuum dried to obtain 117 g of solid powder, with an extraction rate of 23.4%. For chemical characterization, this powder was reconstituted to prepare a stock solution with a concentration of 1 mg/mL (Majida et al. 2025; Kang et al. 2024).

### 2.6 Chemical Characterization of LB Aqueous Extract

The content of total polysaccharides in the lyophilized LB extract was determined using the phenol‐sulfuric acid method with glucose as a standard. Briefly, the extract solution was reacted with phenol and concentrated sulfuric acid, and the absorbance was measured at 485 nm. The polysaccharide content was expressed as glucose equivalents.

The quantitative profiling of specific small molecules was performed using ultra‐performance liquid chromatography coupled with tandem mass spectrometry (UPLC‐MS/MS). Chromatographic separation was achieved on a HYPERSIL GOLD C18 column (3 μm, 2.1 × 100 mm) with a gradient elution of 0.1% formic acid in water (mobile phase A) and acetonitrile (mobile phase B). Mass spectrometric detection was conducted on an AB Sciex Triple Quad 4500 system in multiple reaction monitoring (MRM) mode. Target compounds, including chlorogenic acid, caffeic acid, ferulic acid, isoquercetin, rutin, choline, and betaine, were quantified against their respective calibration curves.

### 2.7 CLP in a Murine Sepsis Model

Sepsis was provoked in 8‐week‐old male C57BL/6 mice (Charles River, Beijing, China) using the CLP method as previously described with modifications (Zi et al. 2024). Briefly, after 12 h of fasting with free access to water, mice were anesthetized via inhalation of 2.5% isoflurane (RWD Life Science, Shenzhen, China). To exteriorize the cecum, a midline laparotomy (1.5 cm) was performed under sterile settings. The distal 75% of the cecum was ligated with 4‐0 silk suture (Jinhuan Medical, Tianjin, China) to prevent obstructing the ileocecal valve. Two through‐and‐through punctures were done using a 21‐gauge needle (BD Biosciences, San Jose, CA, USA), and a little amount of feces was expelled into the peritoneal cavity. The cecum was subsequently returned to the abdominal cavity and the incision closed. Postoperatively, all mice were given a subcutaneous injection of 1 mL of prewarmed saline for resuscitation with fluid. Sham‐operated controls underwent identical procedures except for CLP. After CLP treatment, mice were administered LB dissolved in ultrapure water every 2 days for 6 days. The experimental groups were as follows: (1) mice received ultrapure water (10 mL/kg) after a sham procedure; (2) mice received ultrapure water (10 mL/kg) after undergoing CLP treatment; (3) mice received LB at low, middle and high concentration (100, 300, 500 mg/kg), dissolved in ultrapure water at a volume of 10 mL/kg. For the quercetin treatment, mice were administered it at doses of 25, 50, and 75 mg/kg/day for 7 consecutive days, with an equivalent volume of saline administered to the control group and CLP group. The Ethics Committee of the People's Hospital of Ningxia Hui Autonomous Region ([2021]‐NZR‐098) gave its approval for this investigation. We ensured that all processes were followed in compliance with the applicable norms and legislation.

### 2.8 Cell Culture, Treatment, and Establishment of LPS‐Induced Cardiomyocyte Injury Models

HL‐1 cells (BeNa Culture Collection, China) were cultured in DMEM medium added with 10% FBS, 100 U/mL penicillin, and 0.1 mg/mL streptomycin at 37°C under a 5% CO_2_ environment (Meng et al. 2026). All studies employed cells within passage numbers 4 through 8. First, HL‐1 cells were cultivated to about 80% confluence in 60 mm culture dishes. The HL‐1 cells were subsequently treated for 12 h with 50 ng/mL LPS or saline. To study the effect of LB on LPS‐induced cardiomyocyte damage, HL‐1 cells were exposed to LPS before being treated for 3 h with LB. The setup was divided into three groups: (1) Saline‐treated HL‐1 cell culture, (2) LPS‐treated, and (3) LPS + LB model therapy.

### 2.9 Cell Viability Assessment

The viability of HL‐1 mouse cardiomyocytes was measured with the Cell Counting Kit 8 (CCK‐8) (Beyotime, catalogue number: C0038, Shanghai, China). Briefly, cells were inoculated into 96‐well plates and treated with LPS. CCK‐8 reagent (10 μL) was applied to each well and incubated at 37°C for 2 h. The absorbance at 450 nm was measured (Liang et al. 2026).

### 2.10 RT‐qPCR Assay

Total RNA was taken from the cells of each comparison group separately using a Trizol (Invitrogen, catalogue number: 12183555, Carlsbad, CA, USA) kit. A Reverse Transcription System Kit (Invitrogen, catalogue number: K1691, Carlsbad, CA, USA) was used to create cDNA. RT‐qPCR was performed as recommended using the miScript SYBR Green PCR Kit (Qiagen, catalogue number: 218073, Germany). The data were utilized to examine variations in the relative expression of target genes as 2^−ΔΔCt^ values (Livak and Schmittgen 2001), with GAPDH serving as an internal reference for normalization. Table S2 lists the primers used in the experiment.

### 2.11 Western Blotting Assay

Total protein was extracted from the myocardial tissue of mice and HL‐1 cells using RIPA Lysis Buffer (Epizyme Biotec, catalogue number: PC104, Cambridge, MA, USA, dilution ratio: 1:100); BCA Protein Assay Kit (Epizyme Biotec, catalogue number: ZJ101L, Cambridge, MA, USA) was used to detect cell total protein content. The gel was prepared with a one‐step color PAGE gel rapid preparation kit and electrophoresed until the end. After turning the membrane, using skimmed milk (Epizyme Biotec, catalogue number: PS112L, Cambridge, MA, USA) powder closed for 2 h, a resistance of 2 h incubation, using TBST (Epizyme Biotec, catalogue number: TF103, Cambridge, MA, USA) washing, incubating with secondary antibody for 1 h, resistance with TBST and TBS washing, then enhancement, as we previously reported (Bai et al. 2024). Antibodies to Bcl‐2, Bax, cleaved caspase3, p53, IL‐1β, IL‐6, TNF‐α, ERK, p38, and secondary antibody were purchased from abcam, and the dilution ratio was 1:1000. The target protein‐GAPDH ratio was used to calculate the relative quantity of protein.

### 2.12 Immunohistochemistry Staining

Tissues were processed through standardized histopathological procedures beginning with fixation in formaldehyde, followed by sequential dehydration using graded ethanol series, xylene clearing, and paraffin embedding (Sun et al. 2025). Glass slides were used to mount serial sections that were 4–5 μm thick for the staining procedures that followed. For H&E staining, slices were deparaffinized and rehydrated before being stained with hematoxylin for 5 min to identify nuclei, followed by eosin counterstaining for 2 min to highlight cytoplasmic features. After final dehydration and clearing, slides were mounted with neutral balsam for microscopic assessment of cardiomyocyte morphology and inflammatory cell infiltration. The immunohistochemistry protocol commenced with similar deparaffinization and rehydration steps, followed by endogenous peroxidase blockade using 3% H₂O_2_ for 15 min. Antigen retrieval was accomplished by microwave‐assisted heating in citrate buffer (pH 6.0). Sections were then treated overnight at 4°C with TUNEL primary antibody (Abcam; catalogue number: ab66108, Cambridge, UK; 1:1000 dilution), which was followed by 1‐h room temperature incubation with HRP‐conjugated secondary antibody (Abcam; catalogue number: ab6728, Cambridge, UK; 1:1000). DAB chromogen development proceeded for 5 min with hematoxylin counterstaining to visualize apoptotic cells. After final dehydration and clearing, permanent mounting preceded microscopic image acquisition using standardized digital pathology systems.

### 2.13 Statistical Analysis

All quantitative data were assessed for normality using the Shapiro–Wilk test. Data are presented as mean ± standard deviation (SD). In vivo studies used six mice per group, and in vitro assays were repeated in 3 independent cultures. Statistical comparisons were performed using GraphPad Prism 8 software. For comparisons among multiple groups, one‐way analysis of variance (ANOVA) was applied. When the overall ANOVA indicated significance, Tukey's post hoc test was used for pairwise comparisons. A two‐sided *p* < 0.05 was considered statistically significant for all tests.

## 3 Results

### 3.1 Chemical Composition of the LB Aqueous Extract

The chemical composition of the LB aqueous extract used throughout this study was comprehensively characterized to establish its pharmacodynamic material basis. Quantitative analysis revealed that the lyophilized extract contained 6.659% (w/w) total polysaccharides, as determined by the phenol‐sulfuric acid method, confirming the substantial presence of this major bioactive macromolecular fraction.

Detailed profiling via UPLC‐MS/MS was conducted to identify and quantify specific small‐molecule constituents. A total of seven bioactive compounds were confirmed and quantified. Betaine was identified as the most abundant small‐molecule constituent, aligning with its known prevalence in LB. Other quantified compounds, in descending order of content, included the flavonoid glycosides rutin and isoquercetin, choline, the phenolic acids ferulic acid, chlorogenic acid, and caffeic acid (Table 2). The concurrent presence of these compounds provides a characteristic chemical fingerprint for the extract and supports its multi‐target pharmacological potential. The representative chromatographic profile is provided in Figure S1, and the identification of key compounds was further verified by their primary and secondary mass spectra (Figures S2 and S3).

**TABLE 2:** Quantitative analysis of small‐molecule constituents in the LB aqueous extract by UPLC‐MS/MS.

Compound	Sample dilution factor	Calibration curve	Sample 1 content (μg/mL)	Sample 2 content (μg/mL)
Betaine	10,000	*y* = 132791679.3*x f* + 155676.2467	1706.5541 ± 17.7901	1372.8234 ± 14.1392
Rutin	100	*y* = 11112660.1*x*—121.9372549	72.1904 ± 0.0983	58.2274 ± 0.3999
Choline	100	*y* = 48346538.76*x* + 6780.913026	45.2451 ± 0.5317	39.7742 ± 0.3105
Ferulic acid	100	*y* = 23584783.27*x* + 1229.19548	4.0755 ± 0.0202	3.6868 ± 0.0486
Chlorogenic acid	1	*y* = 29209688.39*x* + 8202.921569	0.5559 ± 0.0019	0.4237 ± 0.0059
Isoquercetin	1	*y* = 3895815.989*x* + 2713.309158	0.2017 ± 0.0015	0.1676 ± 0.0005
Caffeic Acid	1	*y* = 11625060.72*x* + 185.9841457	0.1433 ± 0.0009	0.1171 ± 0.0004

### 3.2 LB Component‐SIMI Target Network Construction

**FIGURE 1:**
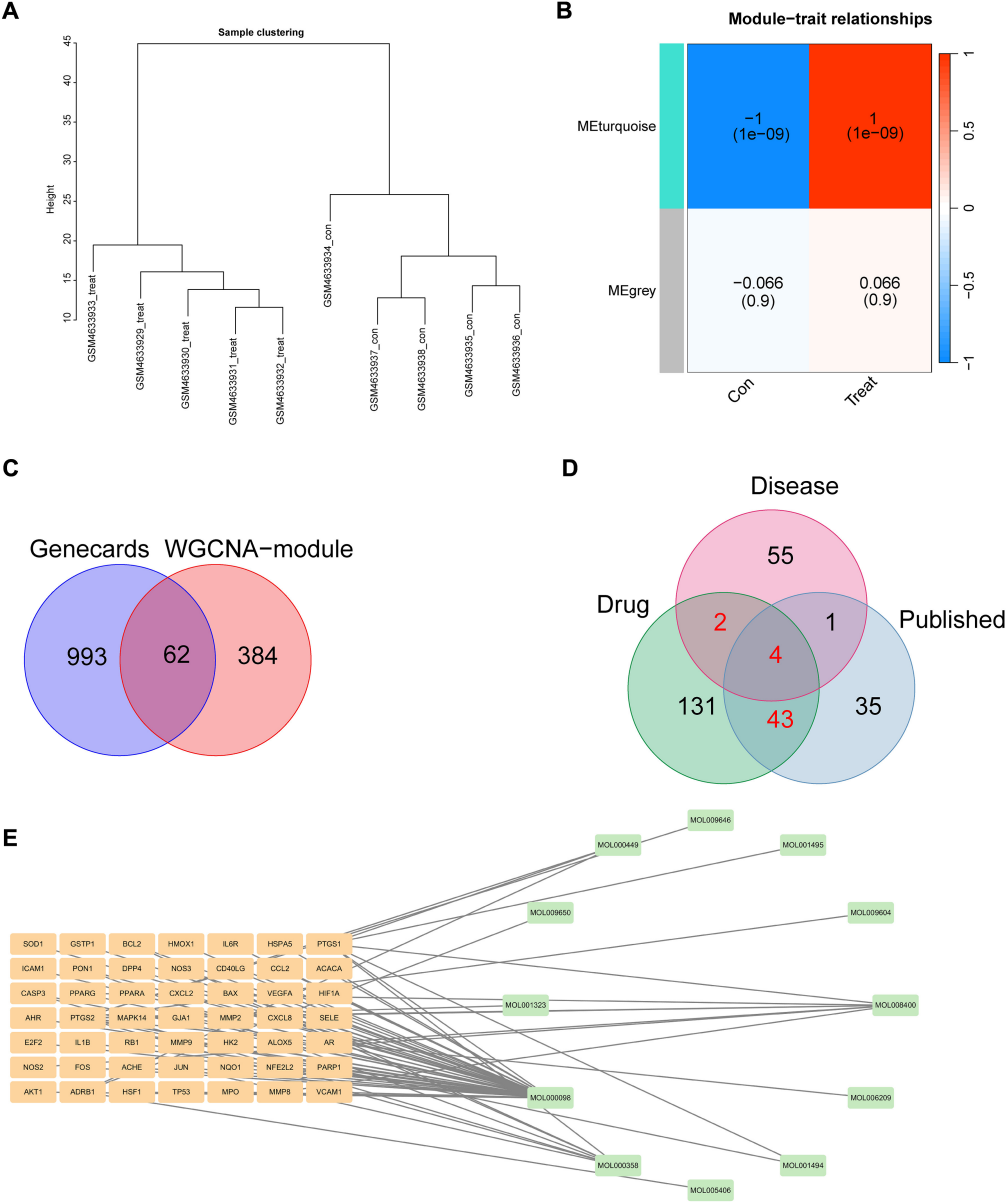
LB component‐SIMI target network construction. (A) Grouped clustering tree diagram for GEO chip data. (B) WGCNA analysis to determine SIMI's most relevant modules. (C) Venn diagram illustrating the identification of 62 candidate SIMI‐related genes. (D) Intersection of LB targets with SIMI‐related genes and reliable genes obtained from PubMed. (E) LB active ingredient‐target network.

TCMSP identified 45 LB components and 320 targets, with OB 30% and DL 0.18 serving as screening criteria (Table S1). To identify SIMI‐associated genes, we first downloaded the corresponding microarray data via GEO. Two groups of 10 samples could be well distinguished by clustering dendrograms (Figure 1A). WGCNA analysis clustered the genes into different modules, and 360 genes in the turquoise module had the highest correlation with SIMI (Figure 1B). Subsequently, we obtained 62 crossover genes from the intersection analysis of Genecards and turquoise module genes as SIMI‐related candidate genes (Figure 1C). By including relevant genes that have been validated by Pubmed and jointly taking the intersection with LB target genes, 49 target genes were obtained (Figure 1D). The LB component‐SIMI target network was created using Cytoscape software, with 49 target nodes and 12 active substance nodes (Figure 1E).

### 3.3 Analysis of the Multi‐Target Network of LB‐SIMI

**FIGURE 2:**
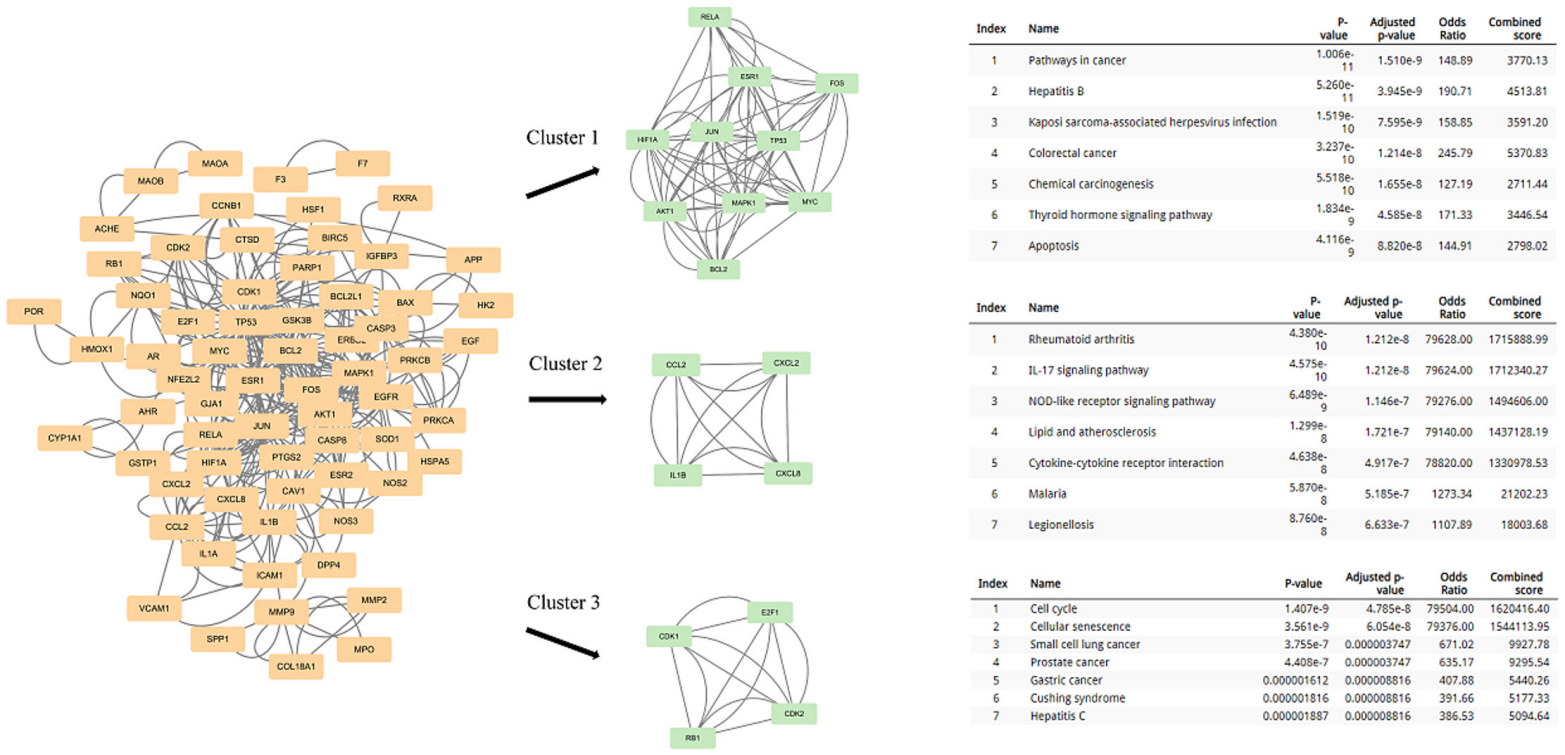
PPI network construction and functional analysis of 49 common targets.

We created a PPI network for the 49 typical LB‐SIMI targets using the STRING database. After setting the confidence threshold to ≥ 0.700, a network containing 210 interaction relationships was obtained, which was visualized using Cytoscape 3.8.2 (Figure 2). The MCODE technique was applied to do a modular analysis of the network. Finally, three functional clusters were identified: Cluster 1 was mostly rich in biological processes such as the regulation of apoptosis; Cluster 2 was significantly associated with the inflammatory response pathway; and Cluster 3 was involved in cell cycle signal transduction (Figure 2). The biological annotation of each functional cluster was accomplished using the EnrichR platform (https://maayanlab.cloud/Enrichr/).

**FIGURE 3:**
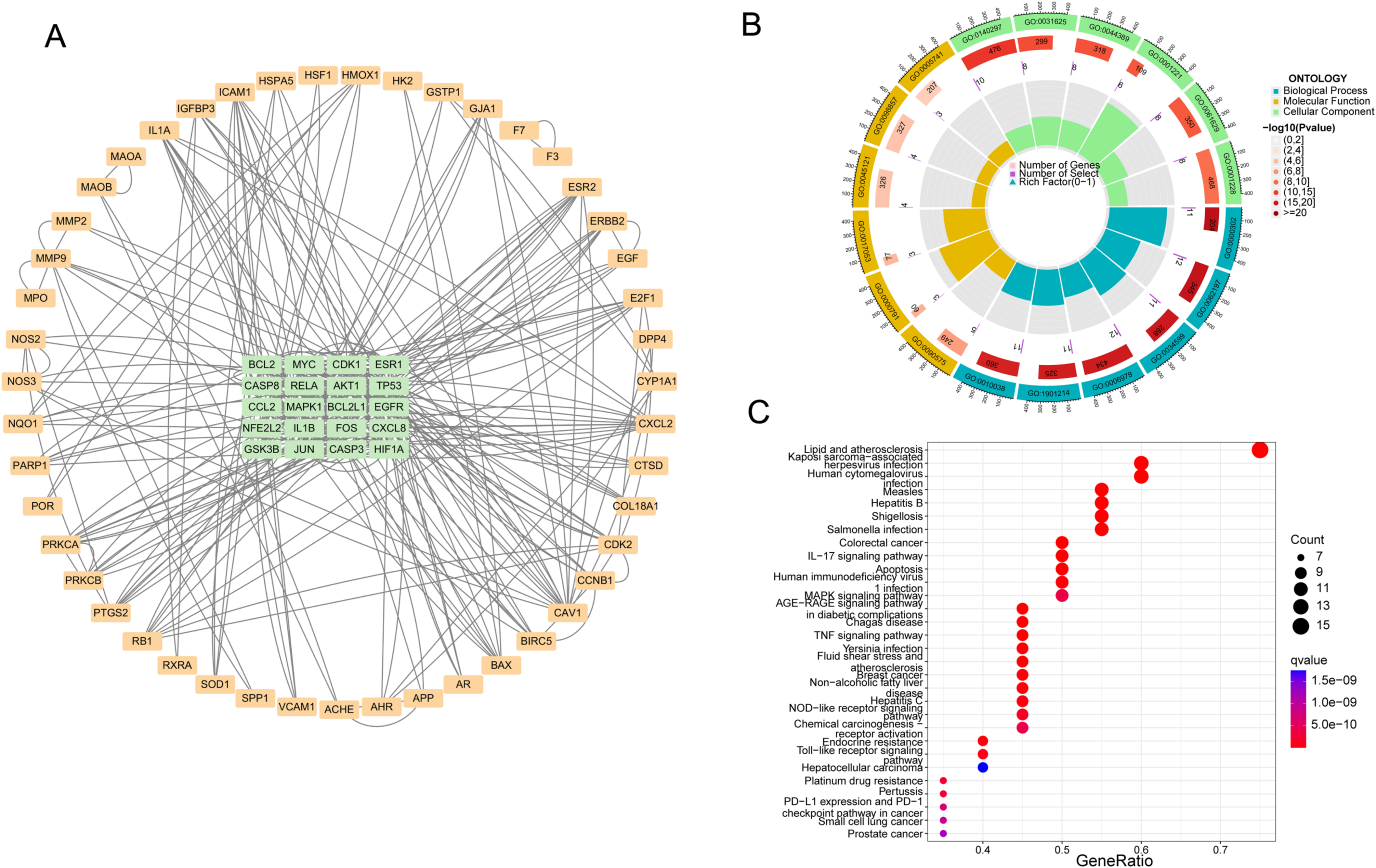
Core protein target screening. (A) PPI network diagram for 20 key protein targets screening. (B) GO and (C) KEGG enrichment analysis of core targets.

Furthermore, the CytoNCA plugin was used to calculate the network topological characteristic parameters, including betweenness centrality (BC), closeness centrality (CC), eigenvector centrality (EC), and local average connectivity (LAC). As shown in Table 1, targets such as TP53, AKT1, and JUN ranked among the top 10% in multiple centrality indices, suggesting their important status as network hub proteins. After screening and obtaining 20 key targets based on the topological characteristics, we reconstructed the core PPI network (Figure 3A). The GO enrichment analysis revealed that these key targets were primarily involved in processes linked to oxidative stress, such as “cellular response to chemical stress” and “cellular response to oxidative stress” (Figure 3B). The KEGG pathway analysis also revealed that they were considerably enriched in the IL‐17 signaling network and the TNF signaling pathway (Figure 3C), implying that LB may exercise its pharmacological effects via modulating cellular oxidative stress and inflammation.

### 3.4 LB Extract Enhances HL‐1 Cardiomyocyte Viability in the Presence of LPS

**FIGURE 4:**
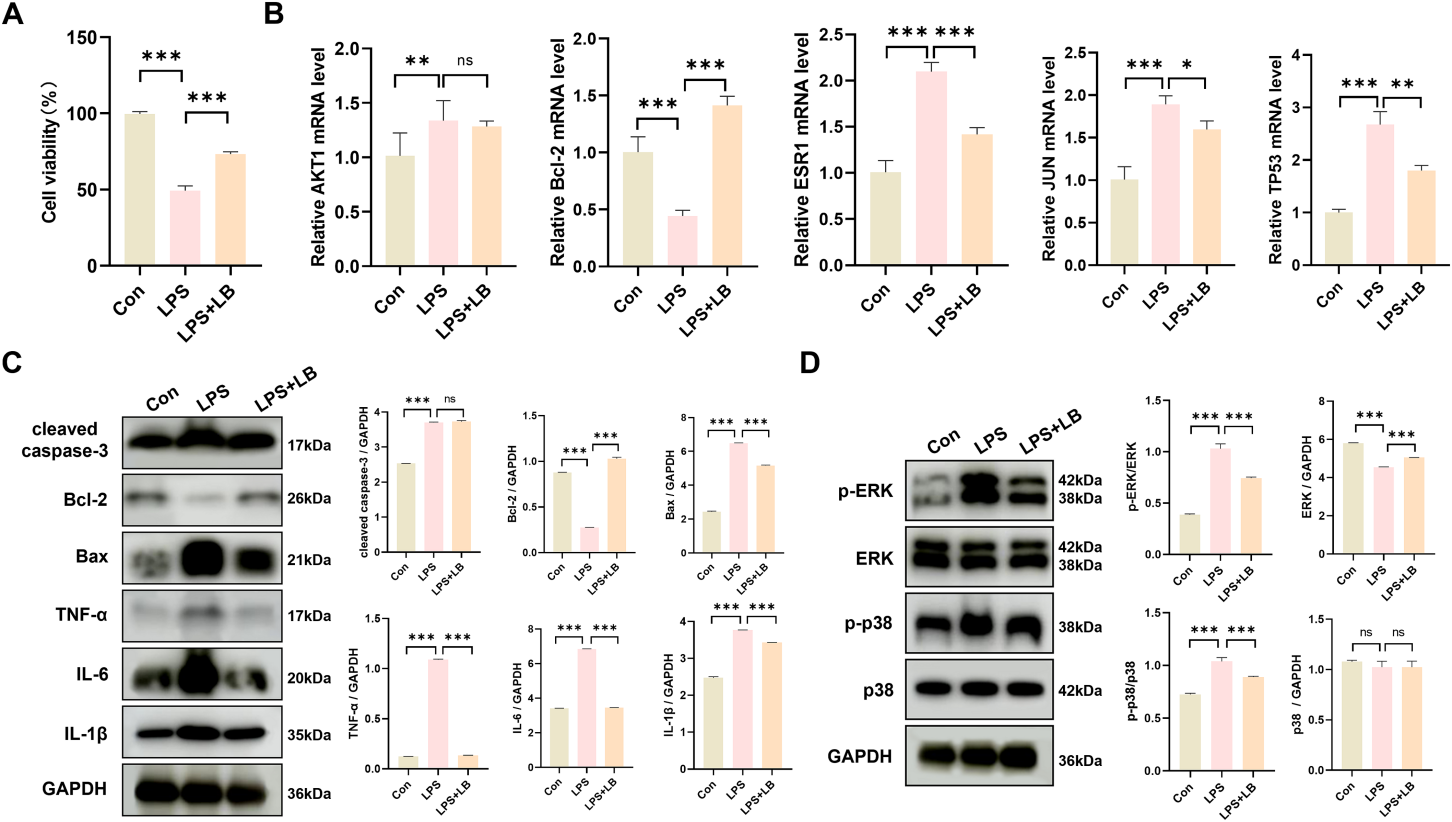
LB inhibits HL‐1 cardiomyocyte viability in the presence of LPS. (A) Cell viability of HL‐1 cells under LPS and LPS + LB treatments showing that LB restored cell viability impaired by LPS (*n* = 3). (B) RT‐qPCR analysis of core targets including AKT1, Bcl‐2, ESR1, JUN, and TP53, with LB reversing the expression changes of most targets induced by LPS (*n* = 3). (C) Western blot analysis of core targets of apoptosis (cleaved caspase‐3, Bcl‐2, and Bax) and inflammation (IL‐6, IL‐1β, and TNF‐α), demonstrating that LB attenuated LPS‐induced increases in apoptotic and inflammatory proteins (*n* = 3). (D) Western blot verified the effect of LB on MAPK pathway‐related proteins in HL‐1 cells (*n* = 3). Data were shown as mean ± SD. Statistical significance was determined by one‐way ANOVA followed by Tukey's post hoc test. *p < 0.05, **p < 0.01 and ***p < 0.001.

To confirm the effect of LB aqueous extract (which contains both polysaccharides and small‐molecule compounds) on the apoptosis of HL‐1 cardiomyocytes in vitro, we exposed HL‐1 cardiomyocytes to LPS and treated them with 1 mg/mL LB aqueous extract. The CCK8 experiment revealed that LPS dramatically reduced cell viability; however, treatment with LB extract reversed this effect (Figure 4A). The qRT‐PCR experiment revealed that the LPS group's mRNA levels of AKT1, ESR1, JUN, and TP53 were dramatically elevated as compared to the Control group, while the Bcl‐2 mRNA level significantly dropped. Following LB extract treatment, all save AKT1 were significantly adjusted back (Figure 4B). Further detection of apoptosis‐related proteins by WB revealed that in the LPS group, cleaved caspase‐3, Bax, and inflammatory factors (TNF‐α, IL‐6, IL‐1β) were significantly increased, while Bcl‐2 was decreased; the treatment with LB extract reversed all these indicators (Figure 4C). Finally, verification of the MAPK pathway revealed that LPS greatly elevated the phosphorylation levels of ERK/p38, which LB extract reversed (Figure 4D). The findings above imply that the LB aqueous extract significantly alleviates LPS‐induced apoptosis and inflammatory response of HL‐1 cardiomyocytes by regulating the MAPK signaling pathway and apoptosis‐related proteins, demonstrating the overall protective effect of the extract containing both polysaccharides and small molecules.

### 3.5 LB Extract Enhances the Viability of Cardiomyocytes in CLP Mice

**FIGURE 5:**
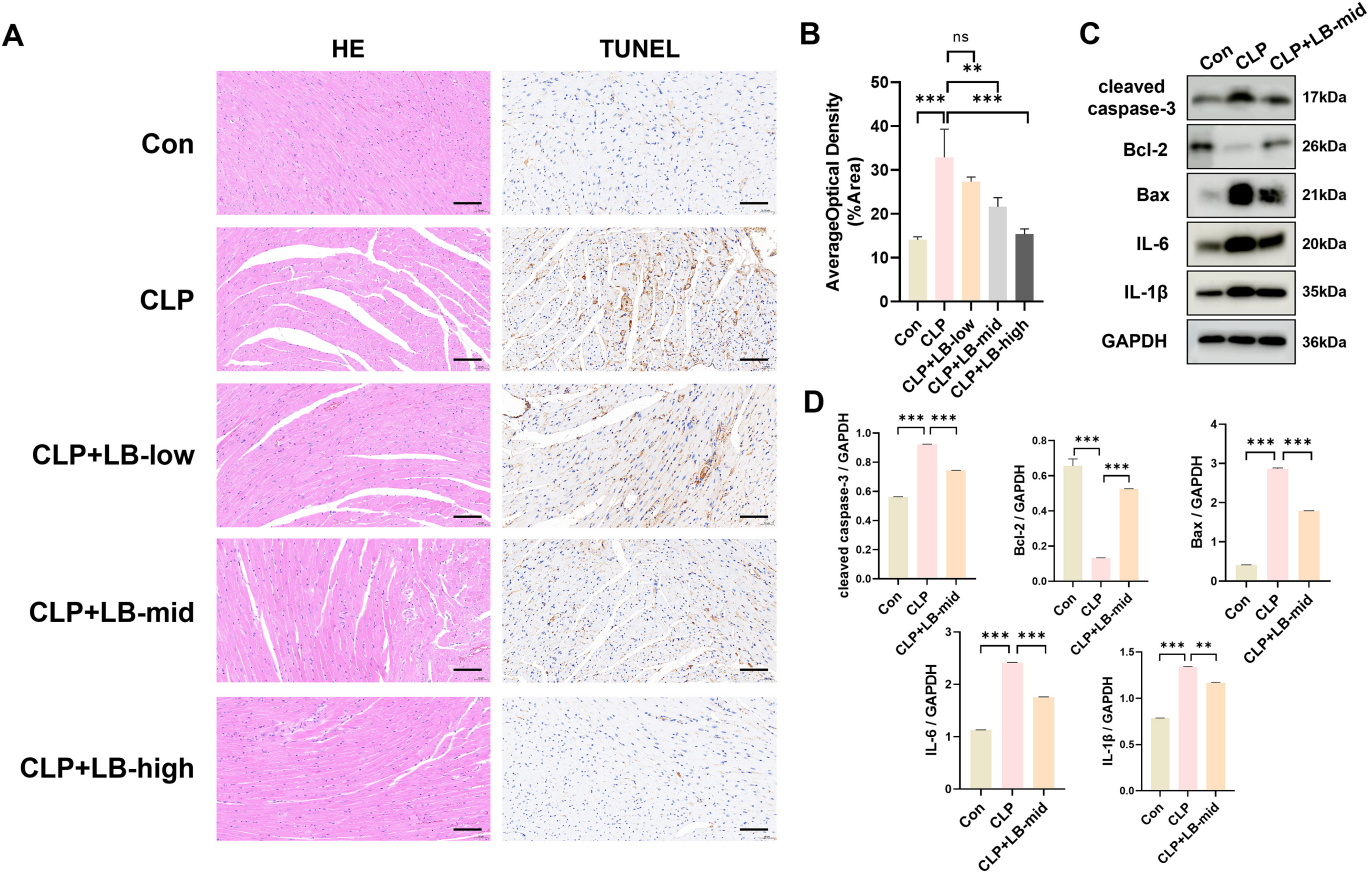
LB protected mice cardiomyocytes after CLP. (A) H&E staining and TUNEL immunohistochemistry of mouse cardiomyocytes, showing that LB alleviated myocardial damage and apoptosis (*n* = 6). (B) Average optical density of TUNEL immunohistochemistry in mice (*n* = 6). (C, D) Western blot and quantification results of inflammatory markers (IL‐6, IL‐1β) and apoptotic markers (Bax, Bcl‐2, cleaved caspase‐3) in mice, showing that LB normalized the expression of key apoptotic and inflammatory proteins altered by CLP (*n* = 6). Data were shown as mean ± SD. Statistical significance was determined by one‐way ANOVA followed by Tukey's post hoc test. *p < 0.05, **p < 0.01, and ***p < 0.001.

H&E and TUNEL staining revealed significant structural abnormalities, myocardial fibrosis, and apoptosis in CLP‐injured myocardium, and LB extract treatment significantly restored these CLP‐induced pathological changes (Figure 5A). Compared to the blank group, immunohistochemistry demonstrated a significant increase in apoptosis within the model group. In a dose‐dependent fashion, LB extract therapy dramatically reduced that increase (Figure 5B). In order to further evaluate the inflammation and apoptosis in mouse myocardium, the markers of inflammation and apoptosis were detected by WB. The findings demonstrated that the protein secretion of cleaved caspase‐3, Bax, IL‐6, and IL‐1β in the model group was significantly higher than in the control group, while Bcl‐2 was significantly lower than in the control group. After LB extract intervention, the protein secretion of cleaved caspase‐3, Bax, IL‐6, and IL‐1β dropped dramatically, whereas Bcl‐2 rose significantly compared to the model group (Figure 5C,D). The findings reveal that in the mouse model of sepsis, the LB aqueous extract can significantly improve the injury of myocardial tissue, indicating the overall protective effect contributed by its constituents.

### 3.6 Molecular Docking Verification of the Interaction Between Key Targets and Active Ingredients

**FIGURE 6:**
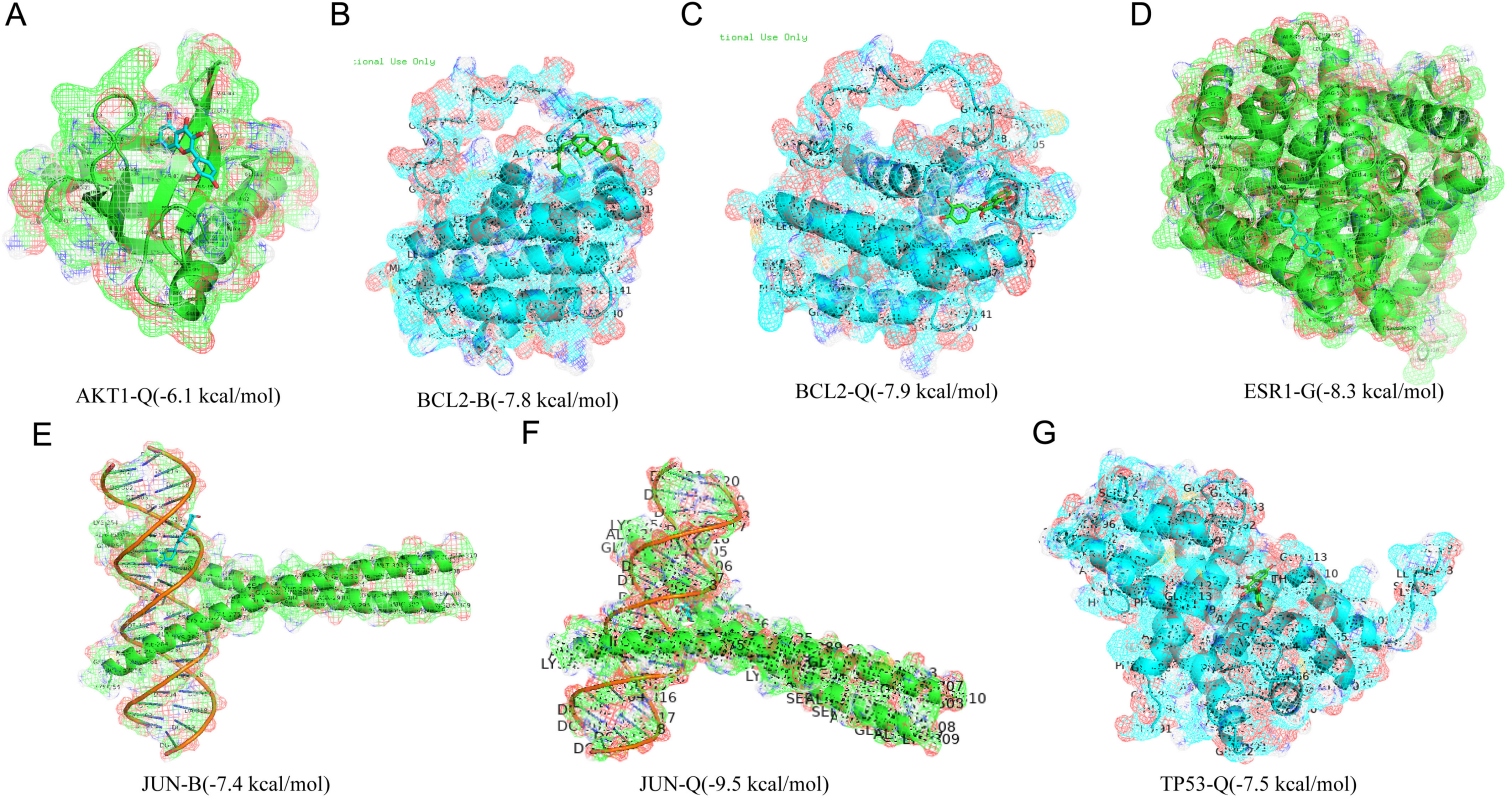
Combination of primary compounds and protein targets including (A) AKT1‐Q, (B) Bcl‐2‐B, (C) Bcl‐2‐Q, (D) ESRD‐G, (E) JUN‐B, (F) JUN‐Q, (G) TP53‐Q. B, β‐sitosterol; G, glycitein; Q, quercetin.

Building upon the confirmed overall efficacy of the LB aqueous extract, we next focused on elucidating the potential contribution of its small‐molecule constituents. To ensure the accuracy of the network pharmacology screening results, we chose major targets (AKT1, Bcl‐2, ESR1, JUN, TP53) with strong topological properties in the PPI network as receptor proteins. According to the screening results of the LB component library (Table S1), we chose the active ingredients closely related to the pathological mechanism of SIMI: β‐sitosterol, quercetin, and glycitein as ligands. Notably, quercetin was selected as it is the aglycone of rutin and isoquercitrin, the flavonoid glycosides quantified in the LB extract, and represents a key bioactive form expected to be liberated in vivo (Anwar and Laila 2024). AutoDock Vina was used to simulate the molecular docking process. As shown in Figure 6A–G, the seven groups of receptor‐ligand pairs all showed stable binding conformations, and their binding free energy ranged from −8.2 to −7.3 kcal/mol, suggesting that these small‐molecule ingredients of LB may exert a therapeutic effect on SIMI by regulating the targets related to apoptosis (TP53/Bcl‐2) and inflammatory response (JUN/AKT1).

**FIGURE 7:**
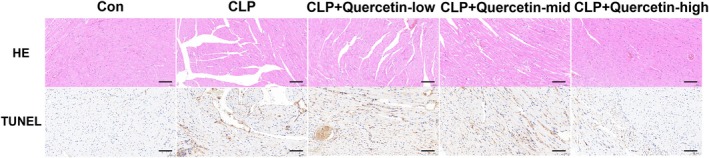
Immunohistochemistry staining showed alleviated SIMI by quercetin treatment, with *n* = 6 in each group of mice.

Among all ligand molecules, quercetin exhibited favorable affinity toward multiple targets; therefore, we subsequently selected quercetin as the primary small‐molecule effector in LB for further validation. To directly test the bioactivity of this predicted compound, we employed exogenously administered quercetin. The results demonstrated that this compound was also capable of ameliorating CLP‐induced myocardial injury in a dose‐dependent manner (Figure 7). In summary, our study demonstrated that LB could significantly alleviate SIMI and further identified that quercetin plays a crucial role in this protection, which was mediated by inhibiting the MAPK signaling pathway, suppressing inflammatory responses, and reducing cardiomyocyte apoptosis.

## 4 Discussion

This study elucidates the multitargeted protective effect of LB against SIMI through an integrated approach. First, experimental chemical characterization of the LB extract was conducted. The bioactivity of LB was predicted to involve binding to core targets such as TP53, AKT1, and JUN. In vitro and in vivo experiments demonstrated that LB inhibits the MAPK signaling pathway, which attenuates cardiomyocyte apoptosis via the regulation of Bax, Bcl‐2, and cleaved caspase‐3, and suppresses the inflammatory response by reducing levels of IL‐6, IL‐1β, and TNF‐α, thereby collectively preserving myocardial structure and function in cellular and animal models of sepsis. Furthermore, quercetin was predicted to be the primary bioactive aglycone derived from the LB extract and was validated in vivo through exogenous administration, thereby confirming its role as a key effector molecule in the observed protection.

### 4.1 The Multi‐Target Mechanisms of LB Extract

Myocardial injury has been recognized as one of the manifestations of sepsis‐induced multiorgan dysfunction (Sun et al. 2022; Xiao et al. 2023). Immune dysfunction and an unchecked inflammatory response are hallmarks of the pathophysiology of sepsis (Nedeva 2021). Despite breakthroughs in therapy and life support, sepsis still has a high death rate (Quan and Gao 2025; Kong et al. 2025). Network pharmacology is a new field that studies the interactions between pharmaceuticals and biological networks by combining systems biology, bioinformatics, and pharmacology (Zhang et al. 2023). Several studies have used network pharmacology to examine the molecular mechanism of different natural compounds, such as notoginsenoside R1 (Shao et al. 2023) and sinomenine (Sun et al. 2023), in sepsis‐induced cardiac damage. In addition to their anti‐inflammatory and antioxidant qualities, these substances may regulate the expression of important genes and pathways that contribute to sepsis‐induced myocardial damage, such as NF‐κB, IL‐17, TNF‐α, and JAK‐STAT (Fu et al. 2022; Liu et al. 2023; Peng et al. 2023). Thus, network pharmacology may provide a useful tool for discovering and elucidating the therapeutic effects of drugs on SIMI.

Water‐soluble active components of LB fruit extracts such as polysaccharides have been reported to possess antioxidant, anti‐inflammatory, and immunomodulatory activities (Zhao et al. 2025). This is consistent with our identification of the IL‐17 signaling pathway and TNF signaling; we found a strong enrichment in apoptotic signaling for LB target proteins (Figure 3C), which focus on the MAPK signaling pathway, and the MAPK signaling pathway is thought to be closely related to apoptosis (Jiang et al. 2025; Zheng et al. 2025). Previous studies have found that excessive inflammation, oxidative stress, and mitochondrial dysfunction in cardiomyocytes undergoing sepsis can activate intracellular apoptotic signaling pathways and further accelerate cardiomyocyte apoptosis (Bi et al. 2022). Our experimental detection of MAPK signaling pathways in myocardial tissue demonstrated that LB exerts therapeutic effects through intervention in the MAPK signaling cascade (Figure 4D). It is noteworthy that IL‐17A, as a key pro‐inflammatory factor, exacerbates myocardial injury by activating the NF‐κB and MAPKs (ERK/p38) signaling pathways (Ge et al. 2020). This mechanism greatly influences the expression of proteins linked to apoptosis (Caspase‐3/Bax/Bcl‐2) and pro‐inflammatory factors (TNF‐α, IL‐6, and IL‐1β) (Huangfu et al. 2023). Western blot detection results further support the idea that LB intervention can have a cardioprotective effect by double‐blocking the inflammatory response and apoptosis (Figure 4C). In the SIMI model, LB intervention significantly down‐regulates the expression of pro‐inflammatory factors and pro‐apoptotic proteins (Bax/Caspase‐3) while simultaneously up‐regulating the level of the anti‐apoptotic protein Bcl‐2 (Figure 5C), which is consistent with its recently reported effects (Wang, Xie, et al. 2026; Wang, Hao, et al. 2026).

A prior study demonstrated that the PI3K/Akt signaling pathway has a strong protective effect on the heart in sepsis‐induced myocardial damage (Wang et al. 2020). Protein kinase activation, including the PI3K‐Akt, has been shown to prevent cardiac myocyte death in cardiac I/R damage (Hausenloy et al. 2011). In our study, while AKT1 mRNA levels remained unchanged in both the model and LB‐treated groups, this observation may indicate that the SIMI model and LB intervention primarily regulate the phosphorylation status rather than the total protein expression of AKT, which has been reported before (Hu et al. 2021). It is important to emphasize that our study elucidates the cardioprotective effect of quercetin, the aglycone of the flavonoid glycosides identified in LB. While our chemical characterization identified rutin and isoquercetin as the predominant flavonoid in the aqueous extract, with no detectable free quercetin, it is well established that dietary flavonoid glycosides undergo deglycosylation in the gastrointestinal tract and are metabolically converted to their corresponding aglycones, such as quercetin, to exert systemic bioactivity (Anwar and Laila 2024; Tamura et al. 2026). This metabolic transformation provides the pharmacological rationale for investigating quercetin as the functional effector in our experimental models, and is further supported by the strong binding affinity between quercetin and the identified core targets (e.g., TP53, AKT1, JUN) as demonstrated by our molecular docking analysis. Notably, other vital components within this botanical have also been established to confer cardiac protection. Emerging evidence indicates that *lycium barbarum* polysaccharide (LBP), a primary active fraction, alleviates SIMI by activating the nuclear factor Nrf2/HO‐1 signaling pathway, thereby suppressing lipid peroxidation and ferroptosis (Niu et al. 2025). Similarly, LBP was shown to inhibit hypoxia/reoxygenation‐induced cardiomyocyte pyroptosis through the Nrf2/HO‐1 signaling pathway (Gao et al. 2024). Furthermore, betaine, another key constituent of LB, has been demonstrated to alleviate SIMI by regulating ferroptosis through the PI3K/Akt signaling pathway (Xiao et al. 2023). These collective findings underscore the pleiotropic nature of LB components in mitigating myocardial injury.

### 4.2 Quercetin as a Key Effector Molecule

In this study, through systematic analysis, we definitively identified quercetin as the primary effector molecule of LB responsible for its therapeutic effect against SIMI, deepening our understanding of LB's multi‐component, multi‐target mechanism at the molecular level. Molecular docking simulations revealed that quercetin exhibited favorable binding affinities toward key SIMI‐related targets, collectively mediating LB's protective effects (Figure 6). These findings not only validated the results of our network pharmacology‐based screening but also provided direct structural evidence for quercetin's role as a bioactive ligand. Notably, while previous studies have reported quercetin's anti‐inflammatory or anti‐apoptotic properties in various disease models (Agrawal et al. 2023; Zhang, Zhang, et al. 2024), this work represents the first explicit positioning of quercetin as the primary effector molecule in LB for SIMI treatment. Traditional Chinese medicine (TCM) research has traditionally concentrated on the overall efficacy of crude extracts, with little investigation into particular active components and their target interactions. Our study fills this gap by combining network pharmacology, molecular docking, and experimental validation, revealing a clear mechanistic relationship between quercetin and LB treatment outcomes. Furthermore, unlike prior investigations that examined quercetin's effects on single pathways in isolation, our research demonstrates its capacity to simultaneously regulate apoptotic and inflammatory signaling cascades, which are features that align closely with the multi‐pathway pathological characteristics of SIMI. This multi‐target regulatory mode may explain why LB confers stronger cardioprotection compared to quercetin monotherapy.

### 4.3 Limitations and Future Perspectives

Despite the integrated approach employed, this study has several limitations. First, while network pharmacology predicted potential targets and pathways, the causal relationships and precise regulatory mechanisms, particularly how LB modulates upstream signaling to affect downstream apoptosis and inflammation, require further mechanistic investigation. Second, the network pharmacology analysis in this study focused on small‐molecule constituents; the potential role of polysaccharides, which are abundant in the LB aqueous extract, was not explored via this computational approach and warrants separate experimental investigation. Third, the in vivo and in vitro models, while established, may not fully recapitulate the complexity of human sepsis. Lastly, although we characterized the major components of the LB extract and validated the protective effect of quercetin as an individual compound, the synergistic or antagonistic interactions among the other multiple bioactive constituents within the complex extract in vivo remain unclear.

Future research should aim to address these gaps. In‐depth studies are warranted to delineate the precise molecular mechanisms by which specific LB components modulate the identified signaling pathways. In addition, dose–response and time‐course studies are needed to quantify the extent of myocardial recovery post‐LB intervention. Furthermore, advancing toward clinical translation will require rigorous pharmacokinetic and toxicity studies of the standardized LB extract, as well as investigation into optimal formulation and delivery strategies to enhance its bioavailability and therapeutic potential for SIMI.

### 4.4 Conclusions

This study used an integrated approach that included network pharmacology, molecular docking, and experimental validation to elucidate the processes underlying LB's therapeutic effects on SIMI. Quercetin, inferred as the bioactive aglycone derived from the quantified flavonoid glycosides (rutin, isoquercetin) in LB, emerged as a compound capable of targeting key proteins AKT1, Bcl‐2, JUN, and TP53. Our findings demonstrate that LB effectively suppresses the expression and secretion of core proteins, attenuates inflammation and apoptosis, and inhibits the MAPK signaling pathway in myocardial tissue of mice, ultimately mitigating myocardial injury. These results highlight the therapeutic potential of LB for SIMI, offering valuable insights for researchers and clinicians seeking effective targeted interventions for this condition.

## Author Contributions

**Yujiao Tang:** methodology, data curation, formal analysis, software. **Ling Zhang:** visualization, conceptualization, validation, writing – original draft. **Weiliang Xue:** methodology, data curation, writing – original draft, validation. **Yi Hu:** conceptualization, data curation, formal analysis, validation. **Xue Bai:** data curation, validation, visualization, investigation. **Liting Yang:** conceptualization, software, validation, visualization. **Zesu Niu:** methodology, data curation, writing – original draft, validation. **Mengfei Chen:** supervision, funding acquisition, project administration, writing – review and editing. **Ruxin Liu:** data curation, validation, software, formal analysis. **Chunxia Ma:** data curation, formal analysis, investigation, visualization.

## Funding

This work was supported by the (Ningxia Hui Autonomous Region People's Hospital Cultivates and Revitalizes Scientific Research Projects) under Grant (202102); and (Ningxia Natural Science Foundation Program) under Grant (2023AAC03457 and 2022AAC03372).

## Disclosure

Generative AI Declaration: No generative AI tools were used during manuscript preparation, data analysis, or figure generation for this study.

## Ethics Statement

This study was approved by the Ethics Committee of the People's Hospital of Ningxia Hui Autonomous Region ([2021]‐NZR‐098 and [2021]‐PYZX‐001). And we confirmed this study is reported in accordance with ARRIVE guidelines.

## Consent

The authors have nothing to report.

## Conflicts of Interest

The authors declare no conflicts of interest.

## Supporting information


**Table S1:** Primer sequences for PCR amplification of the target genes.
**Table S2:** The total available compounds of LB.
**Figure S1:** UPLC chromatographic profile of the *Lycium barbarum* aqueous extract.
**Figure S2:** Representative full‐scan MS1 spectra of the major compounds in the LB extract.
**Figure S3:** Product ion (MS/MS) spectra for the structural confirmation of key constituents.

## Data Availability

The data that support the findings of this study are available from the corresponding author MFC upon reasonable request.
