# Supplementary material for: Mechanisms of Lycium barbarum Treatment in Sepsis‐Induced Myocardial Injury: A Network Pharmacology and Experimental Validation Study
Source: Food Sci Nutr. 2026 Aug 12;14(8):e72197. doi: 10.1002/fsn3.72197 (PMC13463422; doi:10.1002/fsn3.72197)
Supplement: Supplementary file 1 [file FSN3-14-e72197-s001.docx]

**Supplementary information for manuscript “Mechanisms of *Lycium barbarum* Treatment in Sepsis-Induced Myocardial Injury: A Network Pharmacology and Experimental Validation Study”**

**Supplementary Table**

Table S1：Primer sequences for PCR amplification of the target genes

| Gene | Sense primer (5'-3') | Anti-sense primer (5'-3') |
| --- | --- | --- |
| TP53 | CAGCACATGACGGAGGTTGT | TCATCCAAATACTCCACACGC |
| JUN | TCCAAGTGCCGAAAAAGGAAG | CGAGTTCTGAGCTTTCAAGGT |
| BCL2 | GGTGGGGTCATGTGTGTGG | CGGTTCAGGTACTCAGTCATCC |
| AKT1 | AGCGACGTGGCTATTGTGAAG | GCCATCATTCTTGAGGAGGAAGT |
| ESR1 | CCCACTCAACAGCGTGTCTC | CGTCGATTATCTGAATTTGGCCT |

Table S2. The total available compounds of LB

| **Mol ID** | **Molecule Name** | **MW** | **OB (%)** | **DL** | **HL** |
| --- | --- | --- | --- | --- | --- |
| MOL001323 | Sitosterol alpha1 | 426.8 | 43.28 | 0.78 | 5.64 |
| MOL003578 | Cycloartenol | 426.8 | 38.69 | 0.78 | 5 |
| MOL001494 | Mandenol | 308.56 | 42 | 0.19 | 5.39 |
| MOL001495 | Ethyl linolenate | 306.54 | 46.1 | 0.2 | 6.2 |
| MOL001979 | LAN | 426.8 | 42.12 | 0.75 | 5.84 |
| MOL000449 | Stigmasterol | 412.77 | 43.83 | 0.76 | 5.57 |
| MOL000358 | beta-sitosterol | 414.79 | 36.91 | 0.75 | 5.36 |
| MOL005406 | atropine | 289.41 | 45.97 | 0.19 | 2.75 |
| MOL005438 | campesterol | 400.76 | 37.58 | 0.71 | 4.83 |
| MOL006209 | cyanin | 411.66 | 47.42 | 0.76 | 2.72 |
| MOL007449 | 24-methylidenelophenol | 412.77 | 44.19 | 0.75 | 5.1 |
| MOL008173 | daucosterol_qt | 414.79 | 36.91 | 0.75 | 5.6 |
| MOL008400 | glycitein | 284.28 | 50.48 | 0.24 | 16.32 |
| MOL010234 | delta-Carotene | 536.96 | 31.8 | 0.55 | 2.62 |
| MOL000953 | CLR | 386.73 | 37.87 | 0.68 | 4.52 |
| MOL009604 | 14b-pregnane | 288.57 | 34.78 | 0.34 | 4.57 |
| MOL009612 | (24R)-4alpha-Methyl-24-ethylcholesta-7,25-dien-3beta-ylacetate | 482.87 | 46.36 | 0.84 | 8.25 |
| MOL009615 | 24-Methylenecycloartan-3beta,21-diol | 456.83 | 37.32 | 0.8 | 5.67 |
| MOL009617 | 24-ethylcholest-22-enol | 414.79 | 37.09 | 0.75 | 5.32 |
| MOL009618 | 24-ethylcholesta-5,22-dienol | 412.77 | 43.83 | 0.76 | 5.76 |
| MOL009620 | 24-methyl-31-norlanost-9(11)-enol | 428.82 | 38 | 0.75 | 5.49 |
| MOL009621 | 24-methylenelanost-8-enol | 440.83 | 42.37 | 0.77 | 5.43 |
| MOL009622 | Fucosterol | 412.77 | 43.78 | 0.76 | 5.44 |
| MOL009631 | 31-Norcyclolaudenol | 440.83 | 38.68 | 0.81 | 4.88 |
| MOL009633 | 31-norlanost-9(11)-enol | 414.79 | 38.35 | 0.72 | 5.37 |
| MOL009634 | 31-norlanosterol | 412.77 | 42.2 | 0.73 | 5.27 |
| MOL009635 | 4,24-methyllophenol | 414.79 | 37.83 | 0.75 | 4.9 |
| MOL009639 | Lophenol | 400.76 | 38.13 | 0.71 | 4.76 |
| MOL009640 | 4alpha,14alpha,24-trimethylcholesta-8,24-dienol | 426.8 | 38.91 | 0.76 | 6.67 |
| MOL009641 | 4alpha,24-dimethylcholesta-7,24-dienol | 412.77 | 42.65 | 0.75 | 5.44 |
| MOL009642 | 4alpha-methyl-24-ethylcholesta-7,24-dienol | 426.8 | 42.3 | 0.78 | 5.83 |
| MOL009644 | 6-Fluoroindole-7-Dehydrocholesterol | 402.7 | 43.73 | 0.72 | 5.1 |
| MOL009646 | 7-O-Methylluteolin-6-C-beta-glucoside_qt | 318.3 | 40.77 | 0.3 | 14.1 |
| MOL009650 | Atropine | 289.41 | 42.16 | 0.19 | 5.27 |
| MOL009651 | Cryptoxanthin monoepoxide | 568.96 | 46.95 | 0.56 | 4.4 |
| MOL009653 | Cycloeucalenol | 426.8 | 39.73 | 0.79 | 5.01 |
| MOL009656 | (E,E)-1-ethyl octadeca-3,13-dienoate | 308.56 | 42 | 0.19 | 5.47 |
| MOL009660 | methyl (1R,4aS,7R,7aS)-4a,7-dihydroxy-7-methyl-1-[(2S,3R,4S,5S,6R)-3,4,5-trihydroxy-6-(hydroxymethyl)oxan-2-yl]oxy-1,5,6,7a-tetrahydrocyclopenta[d]pyran-4-carboxylate | 406.43 | 39.43 | 0.47 | 7.64 |
| MOL009662 | Lantadene A | 552.87 | 38.68 | 0.57 | 5.44 |
| MOL009664 | Physalin A | 526.58 | 91.71 | 0.27 | 13.21 |
| MOL009665 | Physcion-8-O-beta-D-gentiobioside | 608.6 | 43.9 | 0.62 | 28.46 |
| MOL009677 | lanost-8-en-3beta-ol | 428.82 | 34.23 | 0.74 | 5.48 |
| MOL009678 | lanost-8-enol | 428.82 | 34.23 | 0.74 | 6.41 |
| MOL009681 | Obtusifoliol | 426.8 | 42.55 | 0.76 | 5.91 |
| MOL000098 | quercetin | 302.25 | 46.43 | 0.28 | 14.4 |

**Supplementary Figure**

**Figure S1 UPLC chromatographic profile of the *Lycium barbarum* aqueous extract.**

**Figure S2 Representative full-scan MS1 spectra of the major compounds in the LB extract**

**Figure S3 Product ion (MS/MS) spectra for the structural confirmation of key constituents.**
